# Deep Sequencing Reveals Complex Spurious Transcription from Transiently Transfected Plasmids

**DOI:** 10.1371/journal.pone.0043283

**Published:** 2012-08-16

**Authors:** Jana Nejepinska, Radek Malik, Martin Moravec, Petr Svoboda

**Affiliations:** Institute of Molecular Genetics AS CR, Prague, Czech Republic; The John Curtin School of Medical Research, Australia

## Abstract

Transient plasmid transfection is a common approach in studies in cultured mammalian cells. To examine behavior of transfected plasmids, we analyzed their transcriptional landscape by deep sequencing. We have found that the entire plasmid sequence is transcribed at different levels. Spurious transcription may have undesirable effects as some plasmids, when co-transfected, inhibited expression of luciferase reporters in a dose-dependent manner. In one case, we attributed this effect to a Kan/Neo resistance cassette, which generated a unique population of edited sense and antisense small RNAs. The unexpected complexity of expression from transiently transfected plasmids underscores the importance of appropriate experimental controls.

## Introduction

Transient plasmid transfection is a routine approach to study gene expression in mammalian cells. However, transient plasmid transfections are often used without appropriate attention to potential artifacts. Several factors contribute to this situation. Ancestors of currently used reporter plasmids were developed one or two decades ago (firefly luciferase –1987 [1], *Renilla* luciferase –1996 [2], green fluorescent protein –1994 [3]) when available technologies limited detailed analysis of plasmid expression and its behavior in transfected cells. At the same time, it is difficult to find information about unexpected effects in transfection experiments in the literature. Seminal articles published more than a decade ago [4]–[9] are cited rather infrequently and they are difficult to find among hundreds of irrelevant hits in literature database searches. In addition, the majority of common plasmids used in laboratories are commercial plasmids or their derivatives. Information about these plasmids is often restricted into company’s technical notes (e.g. [10], [11]) while peer-reviewed information about potential problems with these plasmids is limited or even absent.

Reports demonstrating false reporter response in transient transfections suggest three main sources of artifacts. First, reporter plasmids carry artificial cis-regulatory sequences, whose recognition by cellular proteins affects reporter expression at the transcription level [12]–[17]. In addition, recognition of artificial cis-regulatory sequences can be affected by experimental conditions, creating a false treatment response [18]. The second source of problems can be lesions in transfected DNA [7]. The third source of possible artifacts are post-transcriptional regulations. They could involve, for example, expression of complementary RNAs [19] or activation of the protein kinase R (PKR) and the type I interferon (IFN) response [4], [5], [9], [20]. Upon activation by double-stranded RNA (dsRNA), PKR blocks protein synthesis by phosphorylating the eukaryotic initiation factor eIF2 (reviewed in detail in [21]). Taken together, an outcome of a transient transfection experiment can be biased in numerous ways. Yet, published results often include only a minimal set of controls and further details concerning the specificity, reproducibility, dynamic range of the assay *etc*. are not disclosed.

The presented work arose from an internal discussion in the lab regarding which plasmid DNA should be used to maintain a constant amount of transfected DNA per sample. In most cases, one can use a parental plasmid with the same backbone sequence. However, sometimes one needs to select a “neutral” DNA. We previously noticed that different co-transfected plasmids may influence each other. Therefore, we used deep sequencing to characterize transcriptomes of four common plasmids and we further tested sensitivity of luciferase reporters to co-transfection of a larger set of plasmids. We show here that 1) transfected plasmids are a rich source of spurious RNA transcription, 2) pEGFP and its derivatives carrying the same Kan/Neo backbone have strong negative effects on luciferase activity when compared to pBluescript (pBS), and 3) the most likely cause of luciferase reporter inhibition is dsRNA originating from co-transfected plasmids.

## Materials and Methods

### Plasmids

The following plasmids were used in this study: pBluescript II KS (+) (Stratagene), pEGFP-C1 (Clontech), phRL-SV40 (Promega), pCR2.1 (Invitrogen), pJET (Fermentas), pLMP (Open Biosystems), pTMP (Open Biosystems), pSUPER (OligoEngine), pTER [22], and pCAG-EGFP [23]. pGL4-SV40 plasmid was produced by moving SV40 promoter from phRL-SV40 into pGL4.10 (Promega) backbone using BglII-HindIII restriction sites. pRFP-T expressing red fluorescent protein (RFP) was constructed by replacing the EGFP coding sequence in pEGFP-C1 by PCR-amplified monomeric TagRFP sequence (Evrogen) using NheI-BglII restriction sites. pCI-RFPT was constructed by a similar strategy by inserting the TagRFP sequence into the multiple cloning site of the pCI-Neo. To obtain pEGFP_Amp and pRFP_Amp, the kanamycin cassettes (flanked by BspHI restriction sites) in pEGFP-C1 and pRFP-T were replaced by BspHI-released β-lactamase sequence from pCAG-EGFP. Modified plasmids were verified by restriction digest and sequencing.

**Figure 1:**
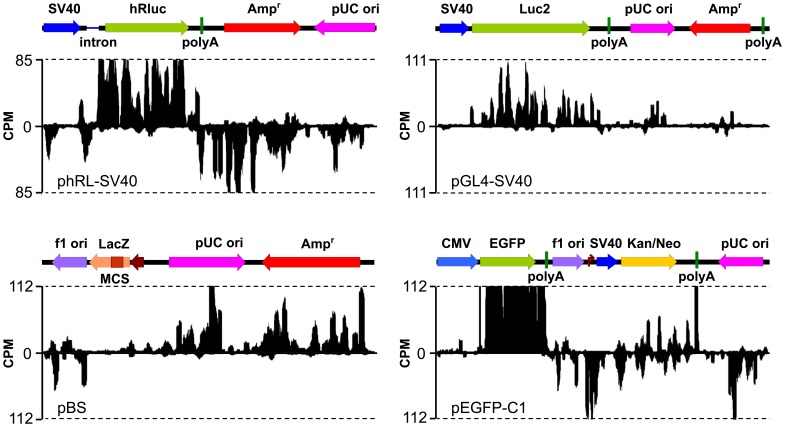
SOLiD sequencing of total RNAs derived from transfected plasmids. Each graph shows the read density along each of the studied plasmids in sense (above X-axis) and antisense (below X-axis) directions. Y-scale represents normalized read density (aligned 18–50 nt reads on a counts per million (CPM) scale). The Y-scale maximum corresponds to 1000 counts of mapped reads and was set to reveal read density in plasmid backbones. Cut-offs for CPM display are indicated by dashed lines. Plasmid annotation is based on maps provided by the manufacturers. Abbreviations: SV40, SV40 early promoter; hRLuc, “humanized” *Renilla* luciferase coding sequence; Luc2, modified firefly luciferase coding sequence; polyA, polyadenylation signal; pUC ori, pUC plasmid replication origin; Amp^r^, β-lactamase open reading frame encoding for ampicillin resistance; f1 ori, f1 single-strand DNA replication origin; LacZ, 5′-terminal part of the lacZ gene encoding the N-terminal fragment of β-galactosidase; MCS, multiple cloning site; CMV, cytomegalovirus promoter; EGFP, enhanced green fluorescence protein; Kan/Neo, Kanamycin/neomycin resistance gene coding sequence. Note that these data offer only an indirect comparison of RNA expression originating from individual plasmids because the amount of an actively transcribed transfected plasmid in a sample cannot be precisely quantified. Source data for all four graphs are provided in the Table S4.

**Table 1:** Deep sequencing results (18–50 nt reads) for different classes of RNAs.

Selected endogenous transcripts							
	length	phRL-SV40		pGL4-SV40		pBS/pEGFP-C1	
	(nt)	RPKM	total	RPKM	total	RPKM	total
***Hprt1***	1,407	**9.78**	776	**9.23**	595	**12.72**	820
***Tbp***	1,844	**3.71**	386	**3.86**	326	**4.44**	375
***Alas1***	2,430	**2.30**	315	**2.44**	272	**2.74**	305
***β_2_-microglobulin***	1,715	**9.04**	874	**7.35**	578	**10.37**	815
***β-actin***	1,917	**21.75**	2,350	**23.09**	2,029	**17.55**	1,542
***γ-tubulin***	1,949	**2.68**	294	**3.27**	292	**2.98**	266
***Gapdh***	1,875	**199.19**	21,055	**201.25**	17,295	**186.88**	16,060
**Selected plasmid-derived transcripts**							
	**length**	**phRL-SV40**		**pGL4-SV40**		**pBS/pEGFP-C1**	
	**(nt)**	**RPKM**	**total**	**RPKM**	**total**	**RPKM**	**total**
**EGFP**	798	**–**	–	**–**	–	**5517.95**	201,821
***Renilla*** ** luciferase**	936	**351.44**	18,544	**–**	–	**–**	–
**Firefly luciferase**	1653	**–**	–	**137.60**	10,425	**–**	–
**Ampicillin CDS**	861	**203.04**	9,855	**30.92**	1,220	**140.26**	5,535
**Kan/Neo CDS**	795	**–**	–	**–**	–	**140.57**	5,122
**Selected microRNAs**							
		**phRL-SV40**		**pGL4-SV40**		**pBS/pEGFP-C1**	
		**RPM**	**total**	**RPM**	**total**	**RPM**	**total**
**miR-19b**		**14.40**	812	**26.49**	1,214	**12.46**	571
**miR-29a**		**17.85**	1,006	**16.25**	745	**17.26**	791
**miR-29b**		**25.86**	1,458	**26.25**	1,203	**21.99**	1,008
**miR-31**		**20.43**	1,152	**17.02**	780	**12.48**	572
**miR-125b**		**50.13**	2,826	**49.92**	2,288	**45.80**	2,099

### Cell Culture and Transfection

Human HEK-293 cells (ATCC no. CRL-1573) obtained from our collaborators [21] were maintained in DMEM (Sigma) supplemented with 10% fetal calf serum (Sigma), penicillin (100 U/mL, Invitrogen), and streptomycin (100 µg/mL, Invitrogen) at 37°C and 5% CO_2_ atmosphere. For transfection, cells were plated on a 24-well plate, grown to 50% density and transfected using Turbofect *in vitro* Transfection Reagent (Fermentas) according to the manufacturer’s protocol. Cells were co-transfected with 100 ng/well of each pGL4-SV40 and phRL-SV40 reporter plasmids and various amounts of the tested plasmid (50–250 ng per well). The total amount of transfected DNA was kept constant by adding pBS. After 48 hours, cells were washed with phosphate-buffered saline (PBS) and lysed with the Passive Lysis Buffer (Promega). Luciferase reporter activity was assessed using the Dual-Luciferase Reporter Assay (Promega) and luminiscence intensity was measured by Modulus Microplate Multimode Reader (Turner Biosystems). Luminometric data were adjusted to the total protein amount in lysates measured by Bradford Protein assay (Bio-Rad) according to manufacturer’s instructions.

### Next Generation Sequencing (SOLiD)

HEK-293 cells were plated on 6-well plates and grown to 50% density. Cells were transfected with pGL4-SV40 (3 µg/well), phRL-SV40 (3 µg/well), or pBS and pEGFP-C1 (each 1.5 µg/well in one well), cultured for 48 hours, washed with PBS, and total RNA was isolated using RNAzol (MRC, Inc.) according to the manufacturer’s protocol. RNA quality was verified by Agilent 2100 Bioanalyzer.

The library construction from total RNA and deep sequencing of RNA transcriptome (RNA-seq) were performed by Seqomics (Szeged, Hungary) using SOLiD (version 4.0) sequencing platform. Bioinformatic analysis was performed as described previously [24]. We used sequence tags (reads) in datasets converted from color-space into the fasta format (fastq output files by Seqomics). The 3′ adaptor sequences were removed from raw reads using an algorithm requiring at least 3-nt exact matches between 50-nt reads and the adaptor sequence.

Sequence reads of 18–50 nt in length (after subtracting the adaptor sequence) were obtained and mapped onto specified plasmid allowing only perfectly matched reads. Each read was allowed to be mapped only once (it was removed from the dataset when it was successfully annotated). Putative polyadenylation sites controlling termination of plasmid-derived transcripts were analyzed as follows: All reads with removed adaptor sequence not aligning perfectly to the plasmid in the first round of mapping were tested for the presence of “A” residue at the 3′ end of the read. When present, all homopolymeric A residues at the 3′ end were trimmed and all resulting read sequences >17 nt in length were mapped on the plasmid. For RNA editing (A>I) analysis, unannotated reads were re-mapped to plasmid sequences allowing for up to five mismatches. Mapping scripts for the analysis were programmed using the Visual Basic 2010 platform (Microsoft).

Sequencing quality and depth of all samples was comparable (Table S1). To confirm that a similar fraction of all reads could be mapped to human genome sequences in all samples, we blasted all reads (original dataset in fastq format) against the human genome (UCSC, built hg18) using memory-efficient short read aligner Bowtie (version 0.12.7) [25] allowing to map reads with up to three mismatches over the entire alignment (using the “V” mode of the Bowtie algorithm). Minor differences in sequencing depths of samples were normalized using the number of reads per kilobase per million mapped reads (RPKM). The RPKM calculation was based on reads perfectly mapping to the genome or the transfected plasmid. We did not find evidence that genome-derived reads would be mistaken for plasmid-derived reads. A control mapping in color-space format was performed to validate results of direct mapping using the fastq format. High throughput sequencing data in color-space format were deposited in the GEO database (GSE36062).

### Flow Cytometry

pCI-RFPT plasmid was labeled using Label IT® Tracker™ Intracellular Nucleic Acid Localization Kit, Cy5 (Mirus) according to the manufacturer’s instructions. HEK-293 cells plated in 24-well plates were co-transfected with 150 ng/well of pCI-RFPT plasmid and 350 ng/well of pBS or pEGFP-C1 plasmid. Cells were collected 36 hours post-transfection and analyzed using LSRII cytometer (BD Bioscience). Data analysis was performed by FlowJo software (Treestar, Inc.). The bleedthrough signal in collected uncompensated FACS data was eliminated by appropriate compensation using controls transfected with only one fluorescent reporter.

## Results

### Deep Sequencing Reveals Complex Expression of Transfected Plasmids

phRL-SV40, pGL4-SV40, pEGFP-C1, and pBS plasmids were selected for deep sequencing. phRL-SV40 and pGL4-SV40 represent common *Renilla* and firefly luciferase reporter plasmids. pGL4-SV40 represents a newer generation of firefly luciferase reporters where putative mammalian transcription factor-binding sites in the plasmid backbone have been extensively mutated to minimize spurious expression [10]. pEGFP-C1 belongs to a family of plasmids for expressing protein fusions with the enhanced green fluorescent protein (EGFP). pBS is a common small cloning plasmid without any annotated eukaryotic transcription unit. All four plasmids utilize pUC prokaryotic origin of replication. phRL-SV40, pGL4-SV40, and pBS carry β-lactamase gene providing ampicillin resistance (Amp^r^) while pEGFP-C1 encodes kanamycin/neomycin (Kan/Neo) resistance for selection in bacteria as well as in mammalian cells.

**Figure 2:**
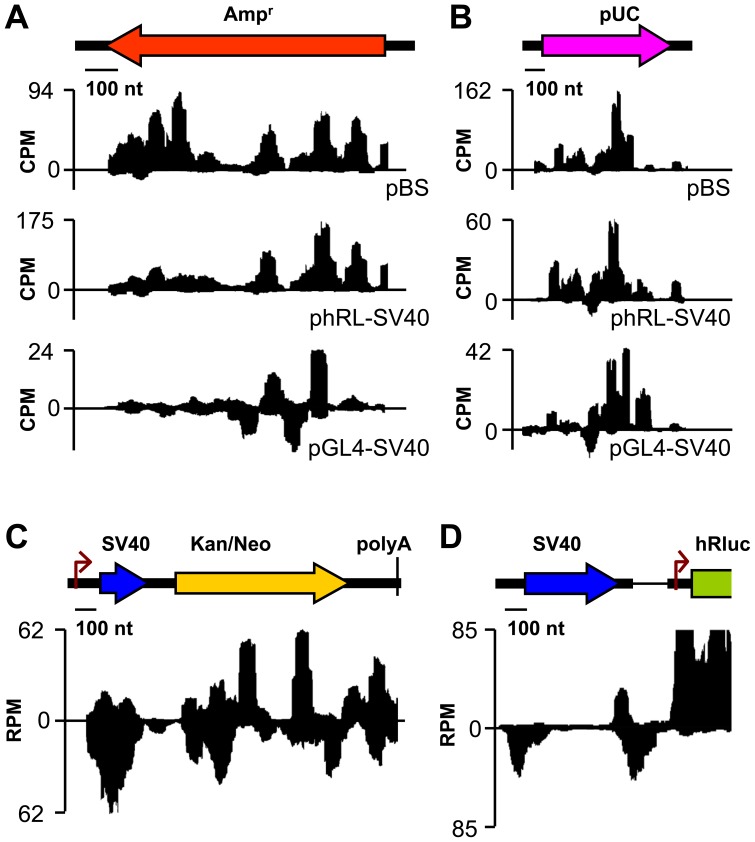
Read densities in specific regions of transfected plasmids. (A) Amp^r^, (B) pUC ori. Note the similar pattern of read density for identical sequences in different plasmids and lower read density and altered patterns in pGL4-SV40. (C) Kan/Neo is the main locus where overlapping sense and antisense expression is detected. (D) SV40 promoter and intron-derived RNAs. Y-scale represents normalized read density – aligned reads on a counts per million (CPM) scale.

Deep sequencing revealed a surprisingly complex picture of transcription originating from transfected phRL-SV40, pGL4-SV40, pEGFP-C1, and pBS plasmids (Fig. 1 and Table 1). Luciferase and EGFP mRNAs were clearly dominating transcriptional landscapes of the reporter plasmids. The most prominent transcript was EGFP mRNA from pEGFP-C1, whose 18–50 nt read frequency was 28,354 RPKM. For comparison, *GAPDH* and β-actin reads were identified in the same sample in 978 RPKM and 91 RPKM, respectively. *Renilla* and firefly luciferase mRNA levels were comparable to endogenous genes (1679 and 699 RPKM, respectively). Strikingly, specific regions in all plasmid backbones yielded read densities within the same order of magnitude as were read densities of Kan/Neo resistance or Firefly and *Renilla* luciferase mRNAs (Fig. 1). These data document that transfected plasmids generate significant amounts of RNA from regions that are not expected to be transcribed in mammalian cells.

**Figure 3:**
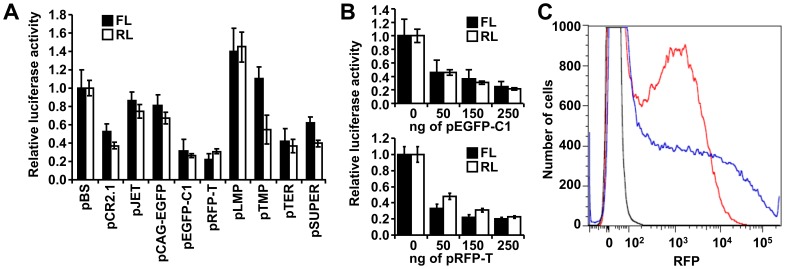
Effects of co-transfected plasmids on expression of luciferase reporters. (A) Different plasmids have different effects on luciferase reporters. HEK-293 cells were co-transfected with 100 ng/well of each luciferase reporter and 150 ng of a tested plasmid. *Renilla* luciferase (RL) and firefly luciferase (FL) activities in pBS co-transfection were set to one. Data represent results of four transfection experiments performed in triplicates. Error bars  =  SEM. (B) Dose-dependent suppression of luciferase activities by co-transfected pEGFP-C1 (upper panel) and pRFP-T (lower panel). HEK-293 cells were co-transfected with 100 ng/well of each luciferase reporter and 0–250 ng/well of pEGFP-C1 or pRFP-T. The amount of transfected DNA was kept constant by adding pBS. Error bars  =  SEM. Data represent results of four transfection experiments performed in triplicates. (C) pEGFP-C1 negatively affects RFP reporter expression. HEK-293 cells were co-transfected with 150 ng/well of pCI-RFPT plasmid and 350 ng/well of pBS or pEGFP-C1 plasmid. RFP expression was analyzed 36 hours post-transfection by flow cytometry. X axis  =  RFP fluorescence intensity. Y axis  =  cell count. Colored curves show distribution of RFP signal as follows: black curve  =  untransfected cells; blue curve  =  pCI-RFPT + pBS co-transfection, and red curve  =  pCI-RFPT + pEGFP-C1 co-transfection. Total counts of transfected (RFP-positive) cells were identical in both samples (Fig. S1C). The shape of the red curve suggests that pEGFP-C1 reduces RFP fluorescence in transfected cells. The experiment has been performed three times, results from a representative experiment are shown.

Plasmid backbone-derived RNAs usually accumulated in distinct clusters in sense or antisense directions, but clusters in opposing directions rarely overlapped (Fig. 1 and 2). The origin and fate of the spurious transcripts remain unknown. While the pattern of read densities would suggest multiple transcription start sites, distribution of reads along reporter mRNAs indicates that the read distribution is also biased by the deep sequencing procedure. Thus, one cannot unequivocally determine transcription start sites. Likewise, termination of the anomalous transcription could not be precisely determined. Analyses of 3′ oligoadenylated sequence tags did not provide conclusive evidence that putative polyadenylation signal sequences are functional. On the other hand, polyadenylation following canonical AAUAAA polyadenylation sites of reporter transcripts was readily detected (data not shown). Expression patterns were typically reproducible for the same sequences. For example, Amp^r^ region yielded RNAs mostly in the antisense orientation relative to the β-lactamase coding sequence and read density along the Amp^r^ or pUC sequences in phRL-SV40 and pBS yielded similar patterns (Fig. 2A, 2B). Even pGL4-SV40, which has a modified backbone sequence, yielded partially similar patterns along the related sequences (Fig. 2A, 2B). The lowest level of spurious transcription was found in the pGL4 backbone, which is modified to have a minimal number of putative transcription factor binding sites (Fig. 1, 2A, 2B). However, we cannot rule out that transfection efficiency contributed to this outcome. The highest level of overlapping sense and antisense transcripts was found in the Kan/Neo resistance gene in pEGFP-C1 (Fig. 2C). A robust antisense transcription originating from the SV40 promoter (Fig. 2C, 2D) and from an intron upstream of *Renilla* luciferase in phRL-SV40 (Fig. 2D) was also remarkable.

**Figure 4:**
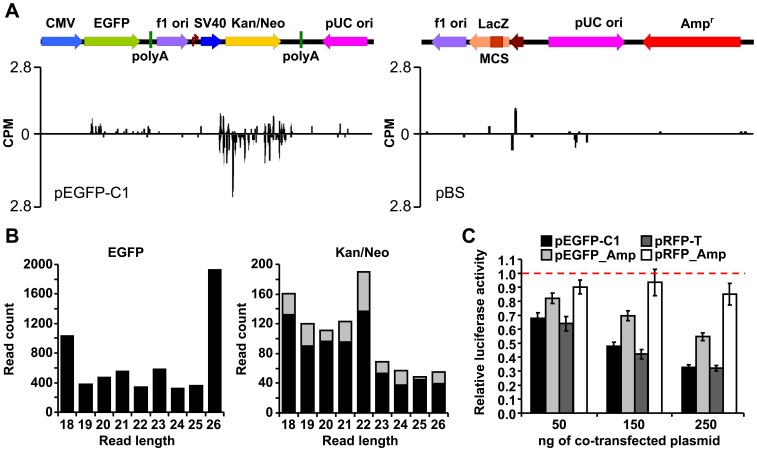
Kan/Neo cassette has a unique small RNA signature and contributes to downregulated expression of luciferase reporters. (A) Analysis of putative adenosine-deaminated small RNAs derived from Kan/Neo cassette (left panel) and pBS (right panel). The distribution of 20–24 nt reads with A/G conversions along pEGFP-C1 and pBS sequences is shown. (B) Size distribution of RNAs originating from EGFP CDS and Kan/Neo CDS sequences in HEK-293 cells. Small RNAs are sorted along the X-axis according to their length (18–26 nt long reads are shown). The Y-axis in both graphs shows the absolute number of reads carrying EGFP- (left) or Kan/Neo-derived sequences (right). The gray portion of each column indicates the fraction of reads carrying up to five A/G sequence changes. Note the absence of edited reads from EGFP CDS region. (C) Replacement of the Kan/Neo cassette by Amp^r^ (denoted by _Amp) relieves repression of luciferase reporters. HEK-293 cells were co-transfected with 100 ng/well of each luciferase reporter and 0–250 ng of one of the four plasmids shown above the graph. The total amount of transfected DNA was kept constant by adding pBS. *Renilla* luciferase activity relative to the sample co-transfected with pBS (dashed line) is shown. Error bars  =  SEM. Data represent two independent experiments done in quadruplicates.

Taken together, deep sequencing analysis of transfected plasmids demonstrated an unexpectedly complex and robust spurious plasmid expression during common transfection experiments. The bulk of the spurious expression occurs reproducibly in specific regions in specific directions. Finally, strong overlaps between abundant sense and antisense expression within individual plasmids are rather uncommon.

### Co-transfected Plasmids Influence Expression

To complement the analysis of spurious transcription from transfected plasmids, we tested how different plasmids influence luciferase reporter expression in co-transfection experiments. We co-transfected ten different plasmids from our plasmid collection with firefly (pGL4-SV40) and *Renilla* (phRL-SV40) luciferases reporters. Tested plasmids represented a diverse set of plasmids used for cloning (pBS, pCR2.1, pJET), as reporters (pCAG-EGFP, pEGFP-C1, pRFP-T), or for RNAi (pLMP, pTMP, pTER and pSUPER). This experiment revealed that majority of plasmids (except for pLMP) inhibited luciferases when compared to pBS (Fig. 3A). Notably, both luciferases were similarly affected suggesting a common mechanism affecting plasmid-derived reporter expression. The strongest repression of luciferase reporters was observed in co-transfection with pEGFP-C1 and pRFP-T plasmids carrying the Kan/Neo resistance gene. The inhibitory effect of pEGFP-C1 and pRFP-T was dose-dependent (Fig. 3B) and was not restricted to luciferase reporters because pCI-RFPT-dependent red fluorescence was also inhibited by co-transfected pEGFP-C1 (Fig. 3C and S1).

Reduction of luciferase activities was apparently not due to cellular toxicity (no reduced growth or increased cell mortality were observed (Fig. S2)). Since pRFP-T exerted the same inhibitory effect as pEGFP-C1, we hypothesized that the inhibition is mediated by the plasmid backbone, which is common for both plasmids, and not by EGFP sequence or its expression. To get further insights into possible causes of inhibitory effects of pEGFP-C1, we re-examined deep sequencing data searching for any transcriptome features unique to pEGFP-C1. The analysis included size distribution of RNA fragments and frequency of A/G conversion (which is a hallmark of edited dsRNA [24]). Consistently with our previous analysis of small RNAs derived from long dsRNA, we have identified a population of RNAs carrying A/G conversions residing within the Kan/Neo cassette of pEGFP-C1 (Fig. 4A). While RNAs carrying A/G conversion were occasionally found elsewhere, none of the other plasmids produced a similar cluster of edited RNAs (Fig. 4A, 4B). Since the Kan/Neo cassette is also a region with a prominent presence of overlapping sense and antisense transcripts (Fig. 1), we conclude that the Kan/Neo cassette generates dsRNA.

To test if the presence of the Kan/Neo cassette is necessary for the inhibitory effect of pEGFP-C1 and pRFP-T on co-transfected plasmids, we replaced the Kan/Neo resistance in pEGFP-C1 and pRFP-T with ampicillin resistance (placed in the same orientation as Kan/Neo). Co-transfection with pGL4-SV40 and phRL-SV40 luciferase reporters showed that the presence of the kanamycin resistance cassette in pEGFP-C1 and pRFP-T plasmids is associated with the reporter inhibition (Fig. 4C). In the case of pRFP-T, exchange of resistance completely abolished the inhibitory effect while there was a residual inhibition observed in the case of pEGFP-C1. This suggests that other factors also contribute to the inhibition by pEGFP-C1 in co-transfection experiments. The relief of repression upon the replacement of the kanamycin cassette was not due to a lower transfection efficiency of ampicillin-bearing plasmids since the number of RFP or EGFP-positive cells remained the same upon resistance exchange (data not shown). Because expression of fluorescent proteins could contribute to inhibitory effects, we analyzed EGFP and RFP fluorescence produced by pEGFP-C1, pEGFP_Amp, pRFP-T, and pRFPT_Amp. Remarkably, cells transfected with pEGFP_Amp exhibited lower EGFP fluorescence while RFP fluorescence of pRFP_Amp was the same as of pRFP-T (Fig. S3). Thus, neither different transfection efficiency nor different expression of a fluorescent protein explains the relief of inhibition when the Kan/Neo resistance is replaced with the ampicillin resistance in pRFP. We propose a model where RNA transcribed from the Kan/Neo sequence in pEGFP-C1 (and likely in other plasmids utilizing the same backbone) inhibits expression of co-transfected plasmids in a process likely involving the formation of dsRNA.

## Discussion

We provide the first deep sequencing-based mapping of transcriptional landscapes of transiently transfected plasmids. Although the level of spurious transcription can be estimated only indirectly, it is clear that it reaches significant levels since read density of spurious transcripts is within the same order of magnitude as that of transcripts produced by annotated eukaryotic genes. Co-transfection experiments suggest that spurious transcription effects depend more on the origin of spurious transcription rather than its level. While pBS and pEGFP-C1 yield comparable levels of spurious transcription, their effects on co-transfected plasmids are in a stark contrast. We cannot rule out that overexpressed CDS in a co-transfected plasmid may also exert inhibitory effects, perhaps related to translational factors consumption. However, the analysis of RFP expression in cells transfected with pRFP-T and pRFP_Amp suggests that a presence of an overexpressed CDS is not the main cause of inhibitory effects on co-transfected luciferase reporters (Figs. S1C and 4C).

The mechanism by which spurious transcription exerts its effect on co-transfected plasmids is unclear at the moment. We ruled out toxic effects on cells or global repression of translation. Some evidence suggests that dsRNA expression triggers the inhibition. First, the Kan/Neo cassette, which is implicated in the inhibition, is the main locus where we observe significant amount of overlapping sense and antisense transcripts (Fig. 2). Second, small RNAs carrying A/G conversions cluster in the Kan/Neo cassette (Fig. 4B). Three lines of evidence suggest that observed A/G conversion is due to the activity of adenosine deamination of dsRNA. First, 21–26 nt-long reads mapping to pEGFP-C1 have the highest A/G nucleotide change rate among all plasmids and among all possible nucleotide changes (Table S2). Second, the editing should result in more frequent appearance of repeated A/G conversions in a single read when compared to random nucleotide changes, which would presumably occur independently of each other in one read. Indeed, A/G conversion was the most frequent repeated nucleotide change in single reads (>80% of all of them) and was mostly found in 21–26 nt-long reads mapping to pEGFP-C1 (Table S3). Third, similar small RNAs were found to be derived from a long RNA hairpin but not single-stranded RNA [24].

It should be noted that our deep sequencing of total RNA does not allow for a good quantitative estimate of the abundance of plasmid-derived small RNAs relative to endogenous mRNAs. While a library preparation causes variable biases [26], we also cannot distinguish, which short tags (<30 nt) existed as small RNAs in cells and which were produced during the library preparation. Also, knowing frequencies of reads matching endogenous miRNAs (Table 1C) does not alleviate the problem since they have a unique origin and their quantitative analysis by deep sequencing is also problematic [27]. Thus, miRNA data may provide an insight into the composition of our deep sequencing libraries but cannot serve as a precise reference point to estimate the abundance of other classes of small RNAs.

dsRNA can induce sequence-specific RNA interference (RNAi) effect. In fact, a study of integrated plasmid repeats in *Drosophila* cells revealed short interfering RNAs (siRNAs) produced from long dsRNA precursors originating from plasmid sequences [28]. We cannot formally exclude RNA silencing because the effect was not directly tested in cells devoid of Dicer. However, it is unlikely that the inhibition of co-transfected reporters involves RNA silencing. First, our previous study of long dsRNA fate in cultured mammalian cells suggests that long dsRNA does not efficiently enter RNAi there [24]. Second, RNAi targets homologous sequences, thus Kan/Neo derived siRNAs could not explain down-regulation of luciferase reporters. Third, a miRNA-like inhibition of luciferase reporters by Kan/Neo-derived siRNAs (i.e. with a less extensive homology), is also unlikely because a large portion of the endogenous gene expression should be strongly affected as well.

It has been reported previously that expression from non-viral and viral vectors is suppressed in mammalian cells in a PKR-dependent manner [9]. Furthermore, while plasmid expression was negatively influenced by the presence of PKR, translation of endogenous proteins was not strongly affected [9]. PKR and dsRNA were also implicated in the selective inhibition of transfected genes in the past [4], [5], [8]. Notably, negative effects of pEGFP-C1 co-transfection are similar to effects of long dsRNA expression in co-transfection experiments (our unpublished observation). At the same time, we have reported that long dsRNA expression has a negligible impact on expression of the genome and does not provoke robust PKR phosphorylation nor the typical type I IFN response to dsRNA [24]. Therefore, we speculate that pEGFP-C1-induced repression of co-transfected luciferase reporters involves PKR-mediated selective inhibition of luciferase expression, which is induced by dsRNA produced by spurious transcription from the Kan/Neo cassette.

Our work has important implications for transient transfection experiments. First, control experiments must be performed to test how different ratios of co-transfected plasmids influence the outcome. Second, experiments should be designed such that they would not require adding of any extra DNA to equalize the amount of transfected DNA. If extra DNA needs to be added, a parental plasmid lacking the “experimental factor” would be the most reasonable choice as it would neutralize the effect caused by the plasmid backbone. However, if one needs a to add a different “neutral DNA”, we recommend using pBS, which has a very common backbone carrying pUC ori and resistance to ampicillin. Third, when combining luciferase and EGFP reporters in one experiment, we recommend avoiding plasmids with the Kan/Neo resistance backbone related to that of pEGFP-C1. Fourth, since several experiments suggest that integrated reporters are insensitive to effects observed during transient transfection [8], [24], a production of stable cell lines should be considered as an alternative or a complementary approach to transient transfections whenever possible.

## Supporting Information

Figure S1 **Co-transfection of pEGFP-C1 inhibits expression of a reporter plasmid without affecting transfection efficiency**. HEK-293 cells were co-transfected with 150 ng of pCI-RFPT reporter plasmid labeled with Cy5 (using Label IT® Tracker™ Intracellular Nucleic Acid Localization Kit, Cy5, Mirus) and 350 ng of either pBluescript (pBS) or pEGFP-C1 plasmid. **(A)** Percentage and **(B)** fluorescence intensity of Cy5-positive cells are similar in pBS and pEGFP-C1-transfected cells demonstrating a similar transfection efficiency. Cy5 fluorescence was estimated as a geometric mean of Cy5 fluorescence intensity shown relative to that of the pBS-transfected sample. Cy5 fluorescence in transfected cells was examined 12 hours post-transfection by flow cytometry. **(C)** Percentage of RFP-positive cells is similar in cells transfected either with pEGFP-C1 or pBS. **(D)** A decrease in RFP-fluorescence in pEGFP-C1 transfected cells indicates an inhibition of a pCI-RFPT reporter expression. RFP expression was analyzed 36 hours post-transfection by flow cytometry. RFP fluorescence was estimated as a geometric mean of RFP fluorescence intensity shown relative to that of the pBS-transfected sample. The experiment was performed three times; the graph shows results of a representative experiment (data from the same experiment are shown in Figure 3C).
(PDF)

Figure S2 **Transfection with pEGFP-C1 or pRFP-T plasmid does not have toxic effects on transfected cells. (A)** Percentage of dead (Hoechst 33258-positive) cells after transfection with different plasmids. HEK-293 cells in a 24-well plate were transfected either with pEGFP-C1 or pRFP-T plasmid (150 ng per well, pBluescript was added to 500 ng per well) or pBluescript (500 ng per well). Cells were analyzed by flow cytometry for the incorporation of Hoechst 33258 dye to visualize dead cells 48 hours post-transfection. Cells treated with Puromycin served as a positive control for Hoechst 33258 staining. There was no increase in a percentage of dead cells in cells transfected either with pEGFP-C1 or pRFP-T plasmids (tested plasmids) compared to Bluescript (pBS)-transfected or untransfected cells. **(B)** Relative protein amount in lysates of transfected cells is not significantly affected by different amounts of EGFP and RFP-expressing plasmids. HEK-293 cells were co-transfected with 100 ng of each phRL-SV40 and pGL4-SV40 reporter plasmids and an increasing amount of indicated plasmid (nanograms of plasmid per well are indicated in parentheses). The total amount of transfected DNA was kept constant by adding pBS. After 48 hours, cells were washed with phosphate-buffered saline (PBS) and lysed in the Passive Lysis Buffer (Promega). Total protein amount in lysates was estimated by Bradford Protein assay (Bio-Rad). Data show a result of a representative experiment performed in quadruplicates. Error bars  =  SEM.
(PDF)

Figure S3 **Effects of replacement of a Kan/Neo resistance cassette by an Amp resistance cassette on reporter expression.** HEK-293 cells were co-transfected with increasing amounts of pEGFP-C1 (GFP-Kanamycin) or pRFP-T plasmid (RFP-Kanamycin) or their derivatives where Kan/Neo^R^ cassette was replaced by Amp^R^ cassette (GFP- and RFP-Ampicillin). The total amount of transfected DNA was maintained constant by adding pBS. The EGFP and RFP fluorescence were analyzed by flow cytometry. Geometric mean fluorescence intensity of **(A)** EGFP-positive and **(B)** RFP-positive cells in a representative experiment is shown. Note that replacement of the resistance cassette in pEGFP-C1 results in a mild reduction of EGFP fluorescence level in EGFP-positive cells while RFP-expressing plasmids yield the same levels of RFP fluorescence regardless of the resistance cassette.
(PDF)

Table S1 **Library metrics.** Reads were mapped using fastq output files from Seqomics as described in Material and Methods.
(DOCX)

Table S2 **Nucleotide change frequencies in transcriptome of cells transiently transfected with selected plasmids.** Reads of 18–50 nt in length were mapped to plasmid sequences allowing for up to 5 mismatches. Frequencies of all possible nucleotide changes were evaluated for short (21–26 nt) or long (50 nt) reads separately. Putative A-to-I RNA editing (represented as A-to-G change) is highlighted in yellow. Frequency of A-to-I RNA editing is at least two-times higher compared to frequencies of other nucleotide changes in short (21–26 nt) reads derived from pEGFP-C1-transfected cells.
(DOCX)

Table S3 **Multiple A-to-G and other conversions within individual reads.** Reads were mapped as indicated in the Table S2. Number of short (21–26 nt) reads mapped with multiple (2–4) identical nucleotide conversions to indicated plasmids is shown in grey. Note the increased number of reads containing multiple A-to-G conversions in pEGFP-C1 sample.
(DOCX)

Table S4 **Figure 1** **CPM source data.**
(XLS)
