# Supplementary figures and images for: Deep Sequencing Reveals Complex Spurious Transcription from Transiently Transfected Plasmids
Source: PLoS One. 2012 Aug 16;7(8):e43283. doi: 10.1371/journal.pone.0043283 (PMC3420890; doi:10.1371/journal.pone.0043283)

**A**

Cy5 positivity

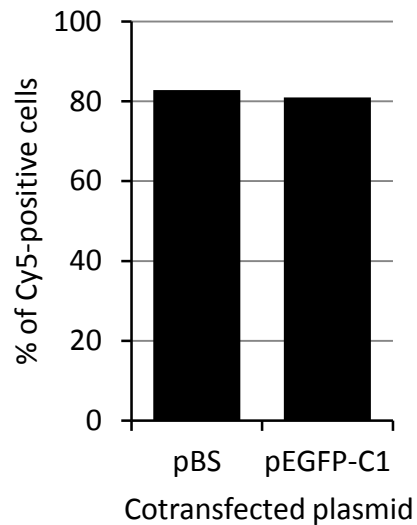**B**

Cy5 fluorescence

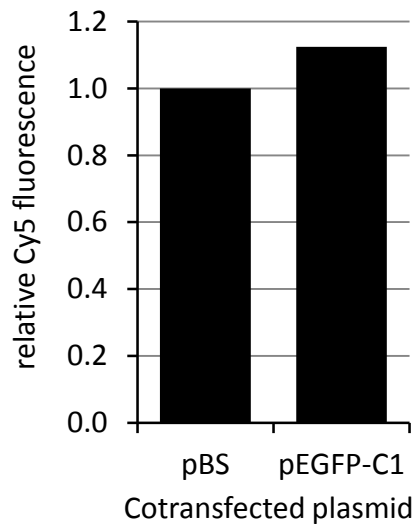**C**

RFP positivity

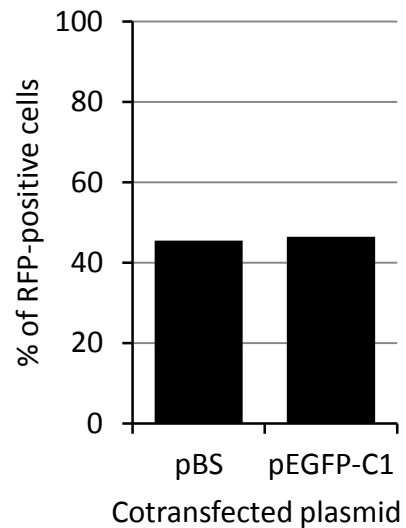**D**

RFP fluorescence

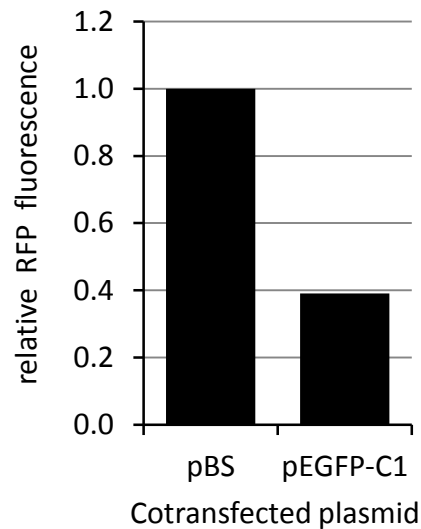

Supplement: Figure S1 — Co-transfection of pEGFP-C1 inhibits expression of a reporter plasmid without affecting transfection efficiency. HEK-293 cells were co-transfected with 150 ng of pCI-RFPT reporter plasmid labeled with Cy5 (using Label IT® Tracker™ Intracellular Nucleic Acid Localization Kit, Cy5, Mirus) and 350 ng of either pBluescript (pBS) or pEGFP-C1 plasmid. (A) Percentage and (B) fluorescence intensity of Cy5-positive cells are similar in pBS and pEGFP-C1-transfected cells demonstrating a similar transfection efficiency. Cy5 fluorescence was estimated as a geometric mean of Cy5 fluorescence intensity shown relative to that of the pBS-transfected sample. Cy5 fluorescence in transfected cells was examined 12 hours post-transfection by flow cytometry. (C) Percentage of RFP-positive cells is similar in cells transfected either with pEGFP-C1 or pBS. (D) A decrease in RFP-fluorescence in pEGFP-C1 transfected cells indicates an inhibition of a pCI-RFPT reporter expression. RFP expression was analyzed 36 hours post-transfection by flow cytometry. RFP fluorescence was estimated as a geometric mean of RFP fluorescence intensity shown relative to that of the pBS-transfected sample. The experiment was performed three times; the graph shows results of a representative experiment (data from the same experiment are shown in Figure 3C). (PDF) [file pone.0043283.s001.pdf]

**A**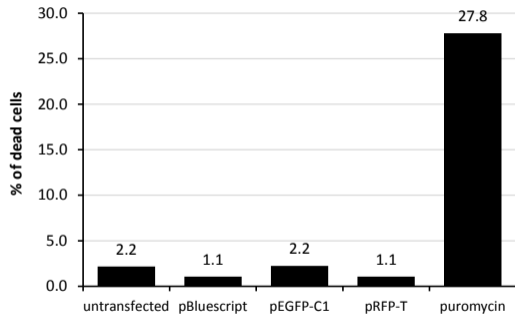**B**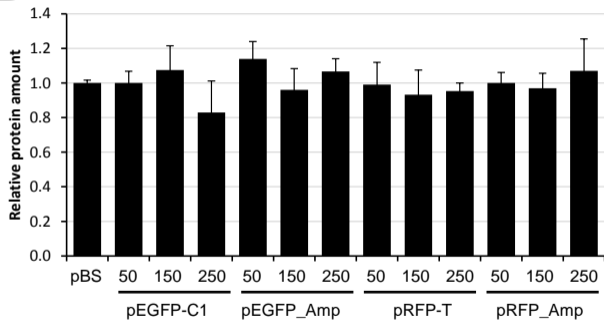

Supplement: Figure S2 — Transfection with pEGFP-C1 or pRFP-T plasmid does not have toxic effects on transfected cells. (A) Percentage of dead (Hoechst 33258-positive) cells after transfection with different plasmids. HEK-293 cells in a 24-well plate were transfected either with pEGFP-C1 or pRFP-T plasmid (150 ng per well, pBluescript was added to 500 ng per well) or pBluescript (500 ng per well). Cells were analyzed by flow cytometry for the incorporation of Hoechst 33258 dye to visualize dead cells 48 hours post-transfection. Cells treated with Puromycin served as a positive control for Hoechst 33258 staining. There was no increase in a percentage of dead cells in cells transfected either with pEGFP-C1 or pRFP-T plasmids (tested plasmids) compared to Bluescript (pBS)-transfected or untransfected cells. (B) Relative protein amount in lysates of transfected cells is not significantly affected by different amounts of EGFP and RFP-expressing plasmids. HEK-293 cells were co-transfected with 100 ng of each phRL-SV40 and pGL4-SV40 reporter plasmids and an increasing amount of indicated plasmid (nanograms of plasmid per well are indicated in parentheses). The total amount of transfected DNA was kept constant by adding pBS. After 48 hours, cells were washed with phosphate-buffered saline (PBS) and lysed in the Passive Lysis Buffer (Promega). Total protein amount in lysates was estimated by Bradford Protein assay (Bio-Rad). Data show a result of a representative experiment performed in quadruplicates. Error bars = SEM. (PDF) [file pone.0043283.s002.pdf]

A

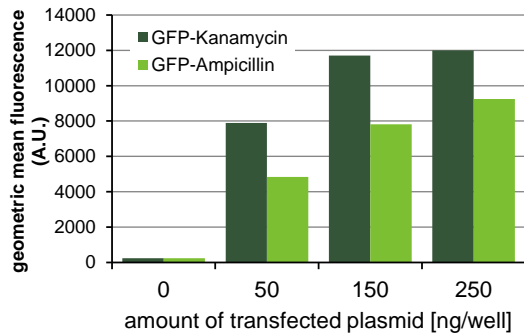

B

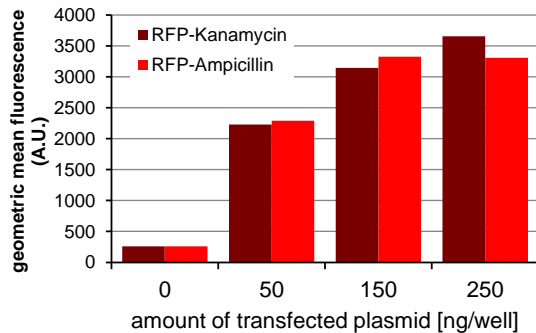

Supplement: Figure S3 — Effects of replacement of a Kan/Neo resistance cassette by an Amp resistance cassette on reporter expression. HEK-293 cells were co-transfected with increasing amounts of pEGFP-C1 (GFP-Kanamycin) or pRFP-T plasmid (RFP-Kanamycin) or their derivatives where Kan/NeoR cassette was replaced by AmpR cassette (GFP- and RFP-Ampicillin). The total amount of transfected DNA was maintained constant by adding pBS. The EGFP and RFP fluorescence were analyzed by flow cytometry. Geometric mean fluorescence intensity of (A) EGFP-positive and (B) RFP-positive cells in a representative experiment is shown. Note that replacement of the resistance cassette in pEGFP-C1 results in a mild reduction of EGFP fluorescence level in EGFP-positive cells while RFP-expressing plasmids yield the same levels of RFP fluorescence regardless of the resistance cassette. (PDF) [file pone.0043283.s003.pdf]
