# Supplementary material for: Deep Sequencing Reveals Complex Spurious Transcription from Transiently Transfected Plasmids
Source: PLoS One. 2012 Aug 16;7(8):e43283. doi: 10.1371/journal.pone.0043283 (PMC3420890; doi:10.1371/journal.pone.0043283)
Supplement: Table S1 — Library metrics. Reads were mapped using fastq output files from Seqomics as described in Material and Methods. (DOCX) [file pone.0043283.s004.docx]

| sample | phRL-SV40 | pGL4-SV40 | pBS/pEGFP-C1 |
| --- | --- | --- | --- |
| number of reads in the library | 56,374,066 | 45,833,776 | 55,581,560 |
| plasmid mapped (perfect) | 41,555 | 15,417 | 251,458 |
| genome mapped reads (perfect) | 11,757,994 | 9,003,682 | 8,668,128 |
| genome mapped reads (1x mismatch) | 3,351,205 | 2,248,047 | 2,420,240 |
| genome mapped reads (2x mismatch) | 1,355,861 | 1,013,057 | 1,030,870 |
| genome mapped reads (3x mismatch) | 1,670,962 | 1,347,401 | 1,240,010 |
| genome mapped reads (all) | 18,136,022 | 13,612,187 | 13,359,248 |
| genome and plasmid mapped (perfect) | 11,799,549 | 9,019,099 | 8,919,586 |
