# Supplementary material for: Deep Sequencing Reveals Complex Spurious Transcription from Transiently Transfected Plasmids
Source: PLoS One. 2012 Aug 16;7(8):e43283. doi: 10.1371/journal.pone.0043283 (PMC3420890; doi:10.1371/journal.pone.0043283)
Supplement: Table S2 — Nucleotide change frequencies in transcriptome of cells transiently transfected with selected plasmids. Reads of 18–50 nt in length were mapped to plasmid sequences allowing for up to 5 mismatches. Frequencies of all possible nucleotide changes were evaluated for short (21–26 nt) or long (50 nt) reads separately. Putative A-to-I RNA editing (represented as A-to-G change) is highlighted in yellow. Frequency of A-to-I RNA editing is at least two-times higher compared to frequencies of other nucleotide changes in short (21–26 nt) reads derived from pEGFP-C1-transfected cells. (DOCX) [file pone.0043283.s005.docx]

| **pGL4-SV40** | 21-26 nt | **A** | **T** | **C** | **G** | 50 nt | **A** | **T** | **C** | **G** |
| --- | --- | --- | --- | --- | --- | --- | --- | --- | --- | --- |
|  | **A** | / | 6.30% | 9.56% | 6.97% | **A** | / | 8.19% | 9.93% | 10.47% |
|  | **T** | 8.62% | / | 7.79% | 5.75% | **T** | 6.05% | / | 6.05% | 5.39% |
|  | **C** | 9.62% | 7.41% | / | 8.07% | **C** | 7.94% | 7.23% | / | 7.90% |
|  | **G** | 9.12% | 9.23% | 11.55% | / | **G** | 10.85% | 10.00% | 9.98% | / |
| **phRL-SV40** | 21-26 nt | **A** | **T** | **C** | **G** | 50 nt | **A** | **T** | **C** | **G** |
|  | **A** | y | 6.93% | 7.55% | 10.44% | **A** | / | 8.60% | 9.36% | 12.77% |
|  | **T** | 9.10% | / | 11.27% | 6.62% | **T** | 6.87% | / | 7.68% | 7.21% |
|  | **C** | 6.41% | 8.69% | / | 8.17% | **C** | 6.22% | 6.89% | / | 7.08% |
|  | **G** | 8.17% | 6.72% | 9.93% | / | **G** | 9.55% | 8.99% | 8.78% | / |
| **pEGFP-C1** | 21-26 nt | **A** | **T** | **C** | **G** | 50 nt | **A** | **T** | **C** | **G** |
|  | **A** | / | 7.67% | 7.01% | **18.90%** | **A** | / | 8.66% | 9.46% | 10.58% |
|  | **T** | 6.32% | / | 6.84% | 6.18% | **T** | 5.31% | / | 5.65% | 5.46% |
|  | **C** | 9.45% | 7.49% | / | 7.32% | **C** | 9.13% | 9.22% | / | 9.54% |
|  | **G** | 8.49% | 6.32% | 8.01% | / | **G** | 9.10% | 8.69% | 9.22% | / |
| **pBS** | 21-26 nt | **A** | **T** | **C** | **G** | 50 nt | **A** | **T** | **C** | **G** |
|  | **A** | / | 6.47% | 6.71% | 10.31% | **A** | / | 9.77% | 9.87% | 13.73% |
|  | **T** | 8.27% | / | 8.15% | 6.83% | **T** | 7.89% | / | 8.26% | 6.63% |
|  | **C** | 8.63% | 7.67% | / | 4.56% | **C** | 5.89% | 6.27% | / | 6.76% |
|  | **G** | 11.03% | 8.75% | 12.59% | / | **G** | 8.78% | 7.75% | 8.38% | / |
