# Supplementary material for: Deep Sequencing Reveals Complex Spurious Transcription from Transiently Transfected Plasmids
Source: PLoS One. 2012 Aug 16;7(8):e43283. doi: 10.1371/journal.pone.0043283 (PMC3420890; doi:10.1371/journal.pone.0043283)
Supplement: Table S3 — Multiple A-to-G and other conversions within individual reads. Reads were mapped as indicated in the Table S2. Number of short (21–26 nt) reads mapped with multiple (2–4) identical nucleotide conversions to indicated plasmids is shown in grey. Note the increased number of reads containing multiple A-to-G conversions in pEGFP-C1 sample. (DOCX) [file pone.0043283.s006.docx]

| identical conversions | | **phRL-SV40** | | **pGL4-SV40** | | **pBS** | | **pEGFP-C1** | |
| --- | --- | --- | --- | --- | --- | --- | --- | --- | --- |
|  |  | A/G | other | A/G | other | A/G | other | A/G | other |
| **2x** | number of reads | **10** | **2** | **0** | **2** | **6** | **2** | **90** | **11** |
|  | % | 83.3 | 16.7 | 0 | 100 | 75 | 25 | 89.1 | 10.9 |
| **3x** | number of reads | **9** | **0** | **3** | **0** | **0** | **0** | **48** | **9** |
|  | % | 100 | 0 | 100 | 0 | 0 | 0 | 84.2 | 15.8 |
| **4x** | number of reads | **0** | **0** | **4** | **0** | **4** | **0** | **44** | **1** |
|  | % | 0 | 0 | 100 | 0 | 100 | 0 | 97.8 | 2.2 |
